# Winning the Confidence of Children

**Published:** 1903-02

**Authors:** Evan P. Wentworth

**Affiliations:** Boston, Mass.


### WINNING THE CONFIDENCE OF CHILDREN.[1]

BY EVAN P. WENTWORTH, D.M.D., BOSTON, MASS.

Mr. President and Fellow-Members of the Harvard Odontological Society,—When a student in the school my first patient was a boy of six years of age. He came to have a cervical filling in the buccal surface of a superior temporary molar. At ten o'clock I began putting on the rubber dam, afterwards excavating the cavity. At twelve o'clock two instructors pronounced it ready to fill. At one o'clock I dismissed the patient, having finished the filling. As you can see, I had some time to think, and so did the patient, who returned the same week for another filling.

From that time I have been much interested in the proper care of children who come to my chair. I bring to you, therefore, as the subject of my paper this evening, "Winning the Confidence of Children."

Experience has taught me to observe that the greatest stoicism or fortitude is sometimes exhibited by very young chlidren, even though their constitutions suffer in consequence; others cry aloud at the least element of discomfort, which is readily elevated by them into a conception of agony.

Other children and certain families are thrown into spasms or convulsions by very little things, whereas others under closely analogous circumstances never manifest any perturbation of the centres of motion. Undoubtedly some children—indeed, some families and races—are relatively insusceptible to pain, and differ even more widely in their capacity to endure.

In my list of patients I find quite a large percentage are children under twelve years of age. Those never having had previous professional services, as a rule, have caused little or no trouble in performing all the operations necessary. Where young patients have been to a fellow-practitioner, and are perfectly satisfied, we seldom hear of them, but some who are not frequently call upon us, oftentimes taxing our patience and ingenuity to the extreme.

It has occurred to me many times, as it probably has to all the members of this Society, that there is a field for our labors before the little patients come to our office. It is well to counsel

---

[1] Read before the Harvard Odontological Society, March 27, 1902.

with the mother concerning her child. She can readily see what is invisible to the most shrewdly observant practitioner. Her testimony is always to be courteously accepted, but not necessarily her conclusions. With some instructions she can aid very much in a previous preparation of the child's mind. She may impart some characteristic which will help in forming an idea of the control she has over it if very nervous, also cause of mental disturbance, if any, when care of teeth is mentioned, all of which may have an important bearing on the situation.

Parents should be induced to take a child early to the dentist, that the first visits at least may be painless. Some children have not the capacity to understand why they are taken right into the dental chair and operations begun without a previous acquaintance, with new surroundings and a stranger dictating to them. Many times they do not submit to the treatment they receive. Some would blame the child, but in reality he is not always at fault.

A patient who has never visited a dentist professionally has no reason within himself to fear the treatment he is about to receive, unless suffering from some pathological condition. Therefore, if only simple operations are to be performed, and he is nervous and scared about something, why is it? I deduce the following principal reason: The operator has not properly led the patient up to the situation, gradually overcoming this fear or that, as fast only as the patient can adapt himself to the circumstances.

When a child comes to meet you, especially if against his wishes, he will look you directly in the eye, and, no matter if he be three or ten years old, before his critical examination is finished decides whether he likes you.

At the first sitting do as little as possible, a simple filling or cleansing, sometimes only an examination, showing the patient the use of some instruments, whether or not the case requires them. If possible accomplish something the patient fears to have done, always dismissing when the best impression is formed and confidence is established. When anything approaching antipathy arises in the child's mind, it must be immediately overcome or a hasty retreat made to some point where he was well satisfied with proceedings.

In some manner pleasing to the patient, on the railroad of the mind the regular train of thought must be side-tracked, while a special is given the right of way until the objectionable features of the operation have been passed.

Allow me to cite a case in my own practice. Patient five years old, with abscessed temporary molar and several simple cavities. I opened the cavity in the abscessed tooth, and put in dressing and cotton sandarach. Stopping there, I dismissed the patient without trying to form any particular impression upon him. At the second visit, when he was not suffering, I was able to interest him with stories, excavate and treat the tooth. At the conclusion of this visit he returned home and appealed to his grandmother for a pail with a handle to it. Upon questioning as to what use he might put it, he said, "Well, I want a pail with a handle and a string." These were brought, and he immediately tied the pail to the left arm of his high chair, and, taking a few steps back, said, "There! that's for folks to spit in." He also got his mother to write a sign on a card, "Robert Blank, Dentist," which he put in his chamber window. Such was his enthusiasm for the profession.

There are practitioners we hear about sometimes who do not favorably impress young children. They cannot depart from the business-like method of handling them as they would any other patient. When a little one, from want of judgment or discretion, should become restless, and even resent some methods of procedure, they still persist as though the operation must be finished in a certain time.

When this patient comes to you for his next treatment he is in a far different state of mind than when he went to visit the above type of operator. You might like to give some expression to your feelings, as a man I once heard of in our late war with Spain. After two days' marching and no food, General Lee's division camped for the night. One of the staff-officers, after vain attempts to sleep with the ground for a bed and a saddle for a pillow, was heard to remark, "Damn that man Columbus for discovering us anyhow."

I have a patient who took her little girl of seven and a half years to a local practitioner to have a tooth filled. According to the mother's description, the child had no fear of the operation and showed none at the treatment received of the family dentist previously. The operator handled her in as matter-of-fact and gruff way as he would an adult who seems never to have his feelings hurt. She showed some uneasiness, and he persisted in the operation, even resorting to scolding as she became more irritable. In a few minutes he had the child in such an hysterical condition that the mother took her to a friend near by. An hour elapsed before the child could be taken home. Now, after two years, when dentistry is mentioned she says, "I'll go and have them pulled, but not filled, mamma."

Another case I have had, similar to the above when it came to my hands. One of my patients told me of a child who was so afraid that the mother could not get her to a dentist. She had visited one and had a filling or two some time previous, but when the matter of a visit to the office was again mentioned, the child got worked up into such a nervous state that the mother gave it up for fear of a very unpleasant experience. She was having trouble with her teeth, and what could be done? I said, "It is time your little girl had her mouth examined;" and, by the way, this child I had seen outside the office several times anticipating that some time she would visit me professionally. To get her in a proper attitude mentally, each time we met I gave her some little attention at the appointed time. This mother with her two children and a neighbor friend, Dorothy, announced themselves at my office. I met them in the reception room. After acknowledging all, I turned my whole attention to my little three-and-a-half-year-old acquaintance. Taking her on my knee, I began talking to her, fully aware I was being watched very critically by her little friend. In a few moments I was talking about a ride, so I took her to the chair, leaving the rest of the party in the reception room. Shortly the mother of my little patient came in and said, "Dorothy remarked, when you left the room, 'Why he don't seem like a dentist!'" This was an expression of what I wished to bring about in the child's mind, and I said, "Let them come in." When my little patient had delighted to ride up and down in my chair I began getting ready to cleanse her teeth. I showed her all the things I used, and tried the revolving rubber disk on her finger-nail. Then I cleaned her teeth, giving her my whole attention, as though no one else was in the room. When this operation was finished, the boy's teeth were examined and cleansed. Then his little friend Dorothy had hers examined and cleansed. She got into the chair while I was preparing for her, and no one had even suggested that she was to have any work done. After the examination and cleansing the company departed. At the subsequent sittings our acquaintance became so intimate, through stories and talks which interested her, that cavities were prepared and filled and all necessary work finished.

Sometimes I have found that a talk on slight of hand, or appealing to the curiosity will serve to place a child in a passive state. A boy who came to me with his brothers and a sister seemed to be the doubting Thomas. As he stood beside the chair I said, "Now, you would be surprised if I should look in your mouth and tell you your name and how old you are." I had never seen him before, but as I was examining his brother's mouth his little sister whispered his name to attract his attention. When he came to the chair I studied his mouth and slowly wrote down his name on my examination blank. He became so mystified that during this sitting and all day until evening his curiosity was so aroused that any thought of my hurting him never seemed to enter his mind, and no trouble was experienced at future sittings.

There are some children who, having had some service rendered them, retain impressions of some objectionable features of the operation. These seem to magnify in the imagination as time elapses. Nothing about the former operation was very painful, yet these worst features loom up in the imagination, so that he shrinks from having attention given the teeth.

It is necessary to go still farther than simply doing the work. Try to place all objectionable features so remote that they will not be retained, at the same time creating an enthusiasm for beautiful teeth.

Whatever the age or intelligence, when the child has not the judgment to control himself, he can usually have his mind occupied with something which most interests him, or information imparted which diverts his attention, so that enough can be accomplished to show that even dental operations may be done and the patient have pleasant memories of the time in which they were performed.

I have called attention to some things that are necessary to the beginning of a child's education regarding the care of the oral cavity. There are many methods of handling children, but they must all be tempered with gentleness, kindness, and patience.

Sometimes I am reminded of a preacher of whom I once heard; not always can I agree with him, however. He had just been blessed by the arrival of a son and heir. His parishioners were to give him a customary donation party. As usual at such gatherings, the exercises were to be opened by prayer. Some of the unregenerates of the congregation made wagers as to which blessing he would speak about first, the new arrival or the many gifts. The prayer ran thus: "O Lord, we thank Thee for this timely succor."

Gentlemen, I thank you for your kind attention.

